# Medial Patellofemoral Ligament Reconstruction in a Below-Knee Amputee

**DOI:** 10.1155/2015/429463

**Published:** 2015-10-22

**Authors:** Sherif El-Tawil, Marian Elfons Tawafig, Jonathan Miles

**Affiliations:** Royal National Orthopaedic Hospital, Brockley Hill, Stanmore, Middlesex HA7 4LP, UK

## Abstract

Patellar instability is a common finding in patients with below-knee amputation and yet management options are not commonly described in the literature. We describe the first reported case of a medial patellofemoral ligament reconstruction using allograft in a patient with a below-knee amputation. Clinical outcome at two-year follow-up remains very good.

## 1. Introduction

Recurrent patellofemoral joint (PFJ) instability can be a disabling condition and is common following initial traumatic patellar dislocation [1]. It is also recognised that patients with a below-knee (BK) amputation have a higher incidence of PFJ instability and postulated reasons include patella alta, trochlear dysplasia, and medial patellofemoral ligament (MPFL) insufficiency [2, 3]. Patella alta is thought to be caused by gradual elongation of the patella tendon by a patella-tendon bearing prosthesis through direct pressure effect [2, 3]. Trochlear dysplasia in patients with BK amputations has been hypothesised to result from reduced use and mechanical loading in flexion of the amputated limb during development in cases where amputations are performed in early childhood [4]. The dysplasia may also directly arise from the same congenital deformity that led to the amputation in the first instance. The altered biomechanics from the use of prostheses can directly place a laterally directed force on the patella during motion, predisposing to lateral subluxation/dislocation, especially in prostheses which are ill-fitting.

MPFL reconstruction has been shown to be an effective way of treating the nonamputee patient cohort with PFJ instability [5]. Rather surprisingly, there has been no study looking at MPFL reconstruction in patients with below-knee amputations, despite its higher incidence in this patient population. There has been a case report of MPFL reconstruction using autograft in a BK amputee [4], but to the best of our knowledge, this is the first report of using allograft to treat PFJ instability in a below-knee amputee.

## 2. Case Presentation

A 25-year-old gentleman presented to the orthopaedic clinic with a three-year history of recurrent patella instability and dislocation in the stump of a BK amputation. The BK amputation was performed in infancy for congenital longitudinal limb deficiency, and the patient had previously experienced excellent mobility with a patella-tendon bearing prosthesis. However, now he reported patella dislocation on an almost bimonthly basis, each time leaving him with pain and swelling that would prevent him from using his prosthesis altogether. Often he would experience so much pain and apprehension that he would not be able to weight-bear on his prosthesis.

On examination he had a well-formed below-knee amputation with a mature stump. Range of motion of the knee was 0–90 degrees with obvious patellar maltracking and moderate quadriceps wasting. Patella apprehension testing was strongly positive.

**Figure 1:**
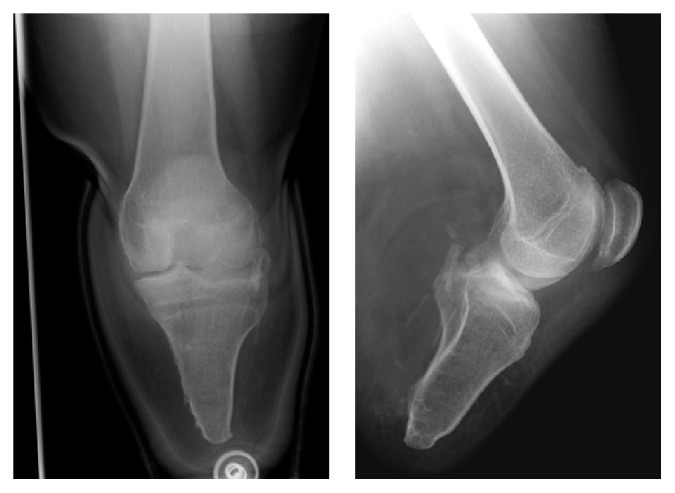
AP and lateral radiographs showing below-knee amputation stump with relative patella alta.

**Figure 2:**
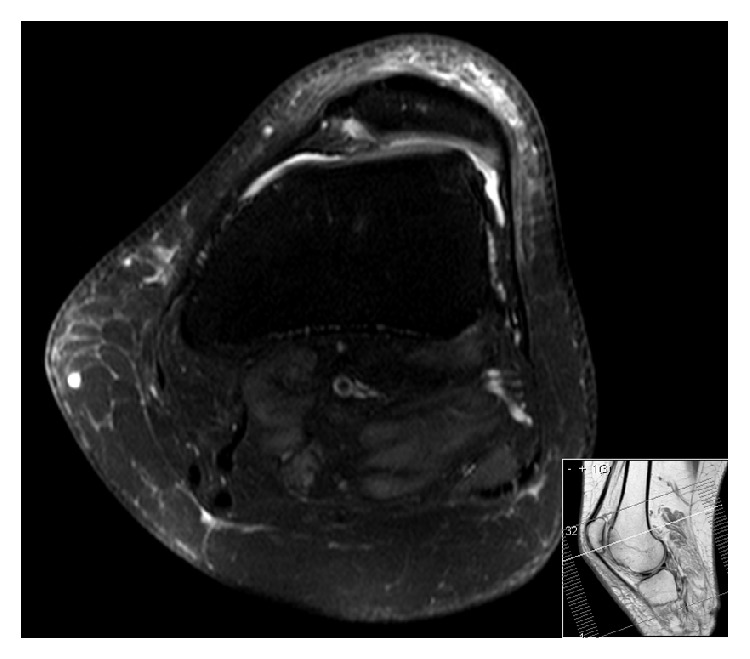
MRI T2-weighted axial image showing a Dejour type C trochlea with lateral condyle convexity and medial condyle hypoplasia.

Radiographs confirmed arthritic changes in the lateral compartment and relative patella alta with an Insall-Salvati ratio of 0.8 calculated according to the original description [6] (Figure 1). Despite having lateral femoral condyle hypoplasia and what initially would appear as distal femoral valgus, the anatomical lateral distal femoral angle measured 88 degrees, which in theory should not encourage patella dislocation. MRI showed severe trochlear dysplasia (Dejour type C) [7] and a laterally displaced and tilted patella (Figure 2). Tibial tuberosity to trochlear groove (TT-TG) distance was 13 mm (within acceptable limits), as measured from MRI. MRI also demonstrated rupture of the MPFL and chondral loss from the medial patellar facet consistent with previous dislocation.

Following a course of failed conservative management with physiotherapy, bracing, and alterations to his prosthesis, the patient elected to undergo MPFL reconstruction. Preoperative MRI showed that the hamstring tendons on the ipsilateral side were atrophied and almost undetectable, and so these were not deemed to be adequate donors. Harvest of ipsilateral quadriceps tendon was not performed for fear of compromising an already compromised limb. The contralateral hamstrings were not harvested as both patient and surgeon did not want to risk any potential complication or weakness in the only functional leg available. As we have easy access to tendon allograft, a semitendinosus allograft was chosen and ordered to reconstruct the MPFL.

## 3. Technique

We used the previously described technique of anatomical double-bundle MPFL reconstruction [8], modified by the use of semitendinosus allograft rather than gracilis autograft. Both ends of the semitendinosus allograft were whip-stitched and diameter measured whilst doubled up. A small incision was placed on the medial border of the patella. With the knee held at 30-degree flexion, a plane was developed between the vastus medialis obliquus (VMO) and the joint capsule, from the medial patellar border to the area of the adductor tubercle where the femoral MPFL insertion is located. A small incision was made at this point just proximal and posterior to the medial epicondyle and a guide wire with an eyelet placed under image intensifier guidance [8]. The wire was then overdrilled up to the contralateral femoral cortex with a drill diameter 1 mm larger than that of the graft loop.

4 mm drill holes (20 mm depth) were placed at the proximal and distal ends of the medial patellar margin, and the two free graft ends were fixed into these using two 4.75 × 15 mm Swivel Lock screws (Arthrex; Naples, FL, USA). The remaining graft loop was then pulled through the plane previously created between the joint capsule and VMO to the femoral insertion. Finally, while maintaining equal tension on both bundles, the graft was pulled into the femoral socket using the guide wire with eyelet and secured using a bioresorbable interference screw.

A lateral release was also performed considering the degree of patella tilt seen on the MRI and clinically.

The knee was immobilised for two weeks in an extension splint and then mobilised fully with physiotherapy. The limb was splinted in extension rather than 30-degree flexion due to the shortness of the stump which would not allow adequate distal hold or stability with a normal hinged brace; we felt that holding the knee in full extension for this short period afforded good graft protection and did not compromise patient rehabilitation or clinical result thereafter. The patient was permitted to use his prosthesis after 3 weeks, although swelling in the stump made this slightly uncomfortable until week 6 postoperatively.

Follow-up at 6 weeks, 6 months, 1 year, and now 2 years showed that the patient had no further episodes of dislocation or symptoms of instability. He had neutral patellar tracking on examination and the patella could not be disengaged with a laterally directed force in full extension. His anterior knee pain fully resolved and he became comfortable using his original prosthesis. Overall, the patient rated his experience as “very satisfied.” He remains aware that the arthritic changes within his knee joint may require arthroplasty surgery in the future but certainly not at present whilst he remains asymptomatic. The patient was also informed of the chondral defect on the medial patellar facet, but this also remains completely asymptomatic.

## 4. Discussion

Patients with BK amputation experience a high incidence of patella instability and dislocation. This has been estimated to be in the region of 25–33% [2, 3]. Yet no study has focussed on the surgical options in this population. Herein we have described a case where MPFL reconstruction using semitendinosus allograft has given good medium term results. We thus demonstrate that the same principles used in the able-bodied cohort can be applied successfully to the stump of a BK amputee despite the significantly altered anatomy and forces arising from the use of a prosthesis.

There has been one case study published that used gracilis autograft from the contralateral knee to reconstruct the MPFL of a BK amputation, also with good effect [4]. Similar to our case, they describe a patient who had a BK amputation in infancy (traumatic, however, compared to our patient who had a congenital longitudinal limb deficiency) and who developed patellofemoral joint instability in later life, associated with patella alta and severe trochlear dysplasia. While we used semitendinosus allograft, they reconstructed the MPFL using gracilis autograft from the contralateral knee, resulting in equally good effect and full patellofemoral joint stabilisation. We preferred to avoid violating the “good leg” in our patient to avoid any potential complications associated with donor site morbidity. However, our decision was also affected by the relative ease of access to allograft in our tertiary unit, which is obviously not widely available in other orthopaedic units.

It is important to recognise that patella-tendon bearing prostheses can elongate the patellar tendon over time, leading to patella alta and PFJ instability. BK amputees can also have trochlear dysplasia resulting from genetic predisposition or the congenital lower limb deficiency itself. The native MPFL is often ruptured from the first episode of traumatic dislocation and is the primary restraint to lateral patellar displacement [9, 10]. There may be concerns that MPFL reconstruction alone would not be sufficient to prevent further instability in such a patient population with such confounding factors as patella alta, trochlear dysplasia, and altered biomechanics arising from the use of a prosthesis. However, as shown in our patient, good results can be attained despite these ongoing concurrent factors that remain unaddressed. Tibial tuberosity transfer was not considered in our patient as the TT-TG distance was within acceptable limits.

Not all units will have access to tendon allograft so it may be necessary to harvest hamstrings from the contralateral knee if suitable. Even ipsilateral harvest can be performed if the pes anserinus remains intact with viable tendon length and size as determined on preoperative MRI, which was not the case in our patient. Thus, we would advocate great attention to preoperative planning to avoid intraoperative dilemmas where no suitable autograft can be harvested and no allograft has been ordered, especially in this patient group with altered anatomy.
